# The challenge continues

**DOI:** 10.20945/2359-3997000000094

**Published:** 2018-10-01

**Affiliations:** 1 Universidade de São Paulo Universidade de São Paulo Faculdade de Medicina Hospital das Clínicas São Paulo SP Brasil Editor-chefe da Archives of Endocrinology and Metabolism, chefe da Unidade de Neuroendocrinologia, Serviço de Endocrinologia e Metabologia, Hospital das Clínicas, Faculdade de Medicina da Universidade de São Paulo (HCFMUSP), São Paulo, SP, Brasil

Four years have elapsed since I took the post of Editor-in-Chief and changed the traditional “*Arquivos Brasileiros de Endocrinologia e Metabologia*” ^1^ to “*Archives of Endocrinology and Metabolism*” (AE&M)^2^. This modification didn't affect the pertinence of the journal to Brazil (it remained the official journal of the Brazilian Society of Endocrinology and Metabolism) but, using English as the official language, became more available to the scientific word. As a matter of fact, the submission of articles from abroad increased substantially, ranging from 44.8% to 52.3% of total, encompassing about 50 different countries, despite the still bigger participation of Brazilian papers. Among the submissions from abroad, the top ten countries were Turkey, China, Iran, Portugal, India, Argentina, USA, Italy, Mexico and Greece, in decrescent order. The amount of total submissions of manuscripts per year (including Brazil) was 306 in 2015, 337 in 2016 and 320 in 2017 (2018 is still ongoing), with an acceptance rate of about 25% of total submissions.

Concerning the impact factor, the marker of a Journal's relevance, it increased from 0.056 as the last “*Arquivos Brasileiros de Endocrinologia e Metabologia*” to 1.076 – the first impact factor of AE&M. This achievement could not be attained without the dedicated and competent collaboration of our Associated Editors, as well as the Editorial Board and all the reviewers. I wish to express a heartfully acknowledgement and gratitude to all of them.

I was honored by my pairs with an additional four years as Editor-in-Chief of the AE&M. I thank them for their confidence and will do my best to accomplish the task to improve our Journal's relevance in the scientific community.
